# Effectiveness of pneumococcal conjugate vaccines against invasive pneumococcal disease among children under five years of age in Africa: A systematic review

**DOI:** 10.1371/journal.pone.0212295

**Published:** 2019-02-19

**Authors:** James Samwel Ngocho, Best Magoma, Gaudencia Alois Olomi, Michael Johnson Mahande, Sia Emmanueli Msuya, Marien Isaäk de Jonge, Blandina Theophil Mmbaga

**Affiliations:** 1 Institute of Public Health, Kilimanjaro Christian Medical University College, Moshi, Tanzania; 2 Kilimanjaro Regional Health Management Team, Moshi, Tanzania; 3 Section Pediatric Infectious Diseases, Laboratory of Medical Immunology, Radboud Institute for Molecular Life Sciences, Radboud University Medical Center, Nijmegen, The Netherlands; 4 Kilimanjaro Clinical Research Institute, Moshi, Tanzania; University of Mississippi Medical Center, UNITED STATES

## Abstract

**Background:**

Despite the widespread implementation of the pneumococcal conjugate vaccine, *Streptococcus pneumoniae* remains the leading cause of severe pneumonia associated with mortality among children less than 5 years of age worldwide, with the highest mortality rates recorded in Africa and Asia. However, information on the effectiveness and prevalence of vaccine serotypes post-roll out remains scarce in most African countries. Hence, this systematic review aimed to describe what is known about the decline of childhood invasive pneumococcal disease post-introduction of the pneumococcal conjugate vaccine in Africa.

**Methods:**

This systematic review included articles published between 2009 and 2018 on the implementation of the pneumococcal conjugate vaccine in Africa. We searched PubMed, Scopus and African Index Medicus for articles in English. Studies on implementation programmes of pneumococcal conjugate vaccine 10/13, with before and after data from different African countries, were considered eligible. The review followed the procedures published in PROSPERO (ID = CRD42016049192).

**Results:**

In total, 2,280 studies were identified through electronic database research, and only 8 studies were eligible for inclusion in the final analysis. Approximately half (n = 3) of these studies were from South Africa. The overall decline in invasive pneumococcal disease ranged from 31.7 to 80.1%. Invasive pneumococcal diseases caused by vaccine serotypes declined significantly, the decline ranged from 35.0 to 92.0%. A much higher decline (55.0–89.0%) was found in children below 24 months of age. Of all vaccine serotypes, the relative proportions of serotypes 1, 5 and 19A doubled following vaccine roll out.

**Interpretation:**

Following the introduction of the pneumococcal conjugate vaccine, a significant decline was observed in invasive pneumococcal disease caused by vaccine serotypes. However, data on the effectiveness in this region remain scarce, meriting continued surveillance to assess the effectiveness of pneumococcal vaccination to improve protection against invasive pneumococcal disease.

## Introduction

*Streptococcus pneumoniae* is a Gram-positive bacterium that asymptomatically colonizes the upper respiratory tract. The colonization rate is 3 times higher in populations living in low and middle income countries (LMICs) (85.0%) compared to those living in high income countries (27.0%) and is higher in children under five years of age compared to adults [1]. Studies have shown that a high colonization rate is a risk for developing an infection. Pneumococcal infections are acquired through aspiration of droplets, leading to pneumonia with or without bacteraemia [2,3]. Approximately 18.0% of all severe pneumonia infections in children less than 5 years of age are caused by *S*. *pneumoniae*, which makes it the second most common cause of severe pneumonia after respiratory syncytial virus. However, it is the leading cause of pneumonia mortality in children less than five years of age (32.7%) [4].

Over 97 different pneumococcal serotypes have currently been identified [5], and their distributions vary widely. The serotype distribution is affected by a number of factors, such as age and geographical location [1]. Worldwide, in the vaccine era, the most common serotype is 14, accounting for 19–26% of all invasive pneumococcal disease (IPD). In LMICs, serotypes 1, 5 and 14 cause more than 30.0% of all IPD [6]. However, in children less than 2 years of age, serotypes 6A, 6B 14, 19F and 23F are the most common, while serotypes 1, 6B, 14, 18C and 23F are the most prevalent in children 2 to 5 years of age. According to Pilishvili and colleagues in the US, serotypes 6, 14, 18 and 19 are the most common serotypes among children below the age of 2 years [7].

Different vaccines have been developed to reduce or eliminate the burden of infections caused by *S*. *pneumoniae*. Currently, two types of vaccines are recommended by the WHO, including the unconjugated 23-valent polysaccharide vaccine (PPSV) and the 10- or 13-valent conjugated polysaccharide vaccine (PCV) [7,8]. The WHO recommends the use of 10- or 13-valent in national immunization programmes, and countries can choose either one of the PCVs to include in their programme, which generally depends on the national epidemiology of *S*. *pneumoniae* serotypes and the cost [1]. The serotypes covered by 10-valent vaccines are 1, 4, 5, 6B, 7F, 9V, 14, 18C, 19F and 23F. In addition to these, the 13-valent vaccine contains serotypes 3, 6A and 19A. These serotypes account for more than 70.0% of all *S*. *pneumonia*-associated IPD based on epidemiological data collected in Western countries [6].

Through the support of the Global Alliance for Vaccination and Immunization (Gavi), most African countries have been able to implement the WHO recommendation of including pneumococcal vaccines in national immunization programmes, with Rwanda being the first African country to roll out PCV13 in 2009. By March 2018, of the 73 Gavi eligible countries 59 (81%) had adopted the WHO recommendation [9]. However, the full roll out is still a challenge to some countries in Africa.

Reduction of pneumococcal carriage and IPD, including pneumonia incidence, caused by vaccine types (VT) has been reported [10]. Inversely, there is evidence of a significant increase in non-vaccine type (NVT) carriage [11]. Some of these NVT have been reported to cause IPD but with a lower-case fatality rate. Furthermore, the protective efficacy against serotype 3 induced by the 13-valent vaccine has been shown to be very limited [12].

The impact of PCV 10/13 on the reduction of IPD and pneumonia is crucial for successful implementation. Although data remain scarce even ten years after the implementation of PCV in Africa, different studies have been conducted in Kenya, Burkina Faso, Morocco, the Gambia, Mozambique, and South Africa, and a clear overview of the current situation is lacking. Therefore, there is a need to gather all available information on the prevalence of VT and on serotype distribution post-roll out of PCV 10/13 in Africa. In addition, surveillance of the highly prevalent serotypes is an important priority.

### Objectives

This systematic review was conducted to estimate the decline of invasive pneumococcal disease among children under five years of age following the introduction of 10 and 13-valent in Africa. In addition, the serotype distribution was compared pre- and post-PCV enrolment.

## Methods

The study protocol development was guided by preferred reporting items for systematic review and meta-analysis, as mentioned in the PRISMA check list (S1 PRISMA check list) [13]. The systematic review protocol was registered in PROSPERO under number CRD42016049192 (available at https://www.crd.york.ac.uk/PROSPERO/display_record.asp?ID=CRD42016049192).

### Eligibility criteria

Studies in children under five years of age conducted in Africa were eligible for inclusion. Additionally, studies that recruited all ages but with stratified data on children less than 5 years of age were included. Studies from countries with either of the two available vaccines (10- and 13-valent) were included, irrespective of the immunization schedules and vaccine coverage.

Publications were excluded based on the following criteria: studies without age-specific data, systematic review articles and studies without data on either pre- or post-PCV introduction. Studies from countries that have yet to roll out PCV and studies without serotype-specific data were not eligible.

### Literature research

We conducted a systematic literature search of published studies on *S*. *pneumoniae* infection. We searched the following electronic databases: PubMed, Scopus, and African Index Medicus (AIM) for studies published between 2009 and 2018. The search was limited to publications in English and used the following key words: *S*. *pneumoniae* carriage, IPD, serotypes, 10-valent and 13-valent vaccine and Africa. We also used the Medical subject heading (MeSH) database to identify synonyms of the subject keywords. The full search strategy is available in the S1 Search strategy (PubMed & Scopus).

### Data collection and quality assessment

**Fig 1:**
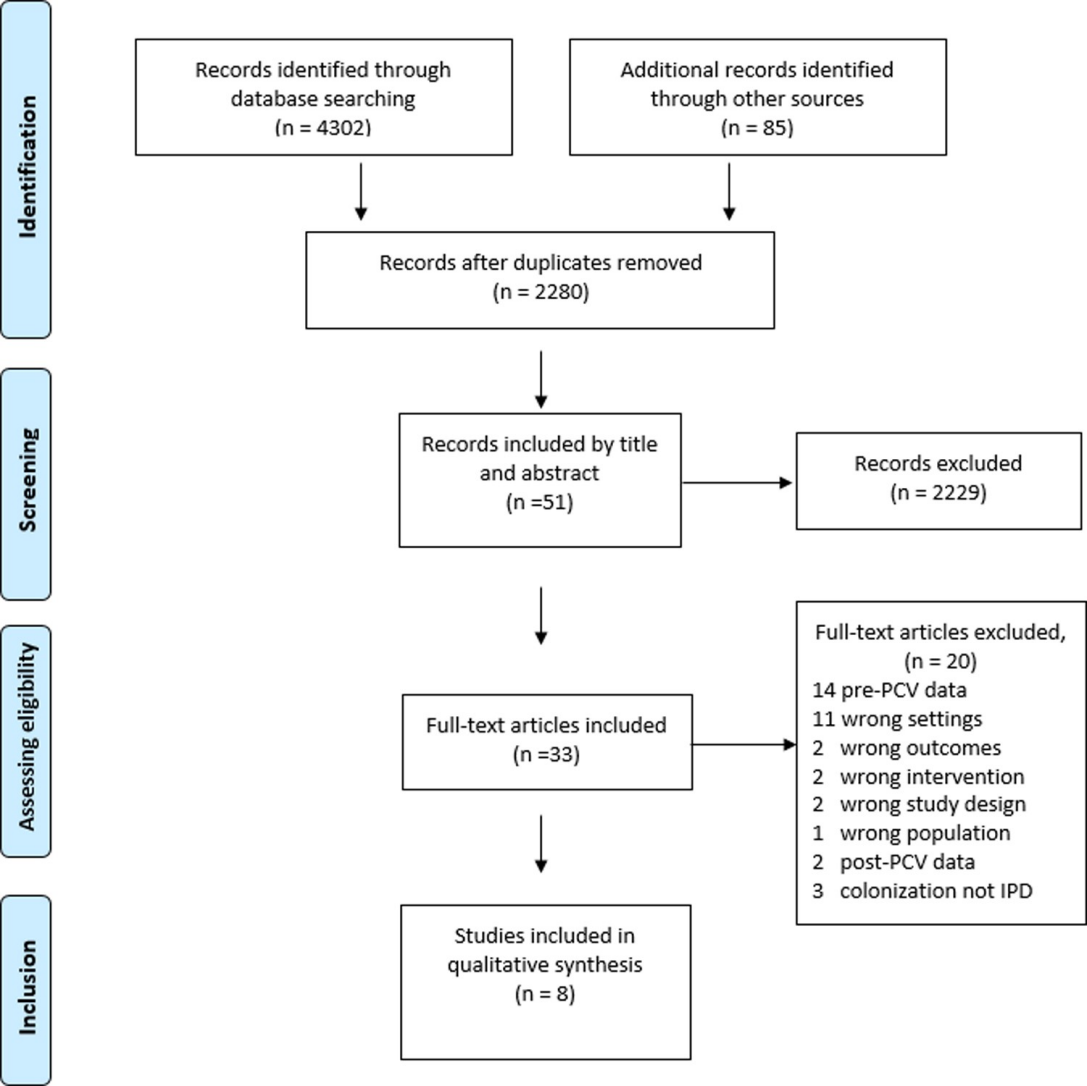
Study selection process.

The results of the searches were all imported into Covidence (https://www.covidence.org/), and JSN performed an automatic check to exclude duplicate entries. The process was followed by screening titles to exclude all irrelevant studies independently by two reviewers (JSN and BTM). In this process of abstract screening, the reviewers either included, excluded or classified abstracts as ‘maybe’. The reviewers met to discuss the disagreements, with a consensus to include or exclude. The same procedure was followed for the full-text screening. The reviewers provided the reasons for excluding studies at this stage. The final included studies for data extraction and the screening process created by Covidence are presented here as part of the PRISMA study flow diagram (Fig 1) [14].

We modified the Cochrane data extraction form [15] and developed an Excel spreadsheet form for this systematic review. We extracted the following information: country, design (start and end date for data collection, duration of participation), study population (eligibility criteria and method of recruitment, total number enrolled, co-morbid infections), outcomes (outcome name, time point measured, outcome definition), and results (comparison, outcome, baseline data, unit of analysis, and method of analysis). Furthermore, we extracted information related to PCV roll out, serotype, country vaccination coverage and participants’ vaccination status.

The risk of bias in cross-sectional studies was assessed using the Newcastle-Ottawa scale (NOS). The NOS is commonly used in the evaluation of evidence in non-randomized studies [16,17]. For studies assessing the effectiveness before and after vaccination, the National Institute for Health checklist was used to assess the risk of bias [18]. A detailed risk of bias assessment is presented in S1 Risk of bias assessment for effectiveness.

### Data analysis

In this review, the primary outcome was IPD caused by vaccine-specific serotypes. We provided a narrative synthesis of the findings because of the significant variations between the included studies regarding the design, vaccine coverage, vaccination status, and outcome measurements as well as with respect to the period between PCV roll out and the start of data collection. There was significant variability in the design and recruitment methods, which made it inappropriate to conduct a meta-analysis.

The vaccine-dependent decline in IPD cases was calculated in two different ways depending on the outcome measure: as the percentage change in the relative proportion of PCV serotypes [(pre-PCV proportion–post-PCV proportion)/pre-PCV proportion [19]] or as the percentage relative risk difference (RR), the ratio of the probability of developing pneumococcal disease occurring in the vaccinated (post-PCV) to the non-vaccinated group (before introduction of PCV), or odds ratio (OR) before and after vaccination. The decline was calculated using the formula: 1-OR or 1- RR [19].

## Results

### Identification of eligible PCV vaccination studies

In total, 2,280 research articles were identified during the electronic database search. Eight research articles were eligible for inclusion into the final analysis (Fig 1). Of the eight included articles, three were from South Africa. Of the included countries, three used the 13-valent vaccine, while three countries used the 10-valent vaccine. Five of the included studies had no data collection interruption during the study period starting the year before and extending to the years after the introduction of the vaccine. Six of the included studies assessed the serotypes using routinely collected samples for pneumococcus surveillance (Table 1) [20–23].

**Table 1:** Characteristics of the studies included in the review.

Author	Country	PCV	Introduction year	Reported coverage %	Study period	Study design	Identification method
Von Gottberg et al., 2014 [21]	South Africa	13	PCV7–2009 PCV13–2011	2009–10 2012–81	Before: 2005–2008 After: 2011–2012	Before and after vaccine Laboratory surveillance	Culture
Diawara et al., 2015 [22]	Casablanca, Morocco	10	PCV13–2010 Pcv10–2012	2014–88	Before: 2007–2010 After: 2011–2014	Before and after vaccine Laboratory surveillance	Culture
Mackenzie et al., 2016 [24]	The Gambia	13	August 2009 –PCV13 June 2011 –PCV10	2010–35 2013–94	Before: 2008–2010 After: 2013–2014	Before and after vaccine Population surveillance	Culture
Von Mollendorf et al., 2016 [27]	South Africa	13	PCV7–2009 PCV13–2011	2009–10 2013–62	Before: 2003–2010 After: 2011–2013	Before and after vaccine Laboratory surveillance	Culture and PCR
Tempia et al., 2015 [26]	Soweto, South Africa	13	PCV7–2009 PCV13–2011	2009–10 2012–81	Before: 2009 After: 2011–2012	Before and after vaccine Laboratory surveillance	Culture and PCR
Nhantumbo et al., 2017 [25]	Mozambique (3-regions)	10	March 2013	97	Before: 2013 After: 2014–2015	Before and after vaccine Laboratory surveillance	Culture and PCR
Hammitt et al., 2018 [28]	Kilifi Kenya	10	January 2011	2011–80 2017–84	Before: 1999–2010 After: 2012–2016	Before and after vaccine hospital surveillance	Culture, latex & quelling reaction confirmed by PCR
Kambire et al., 2018 [23]	Burkina Faso	13	October 2013	2015–105%	Before: 2011–2013 After: 2014–2015	Before and after vaccine Laboratory surveillance	Culture and latex 93.4%-confirmed by PCR

### Decline in IPD caused by vaccine serotypes

Several studies reported the decline of IPD by age categories [21–26], while von Mollendorf and colleagues reported an overall vaccine decline among children under the age of 5 without age stratification data on the age category [27]. Von Gottberg and colleagues reported disease reduction due to vaccine types, covered by 7- and 13-valent [21]. Diawara et al. (2015) conducted the same analysis and reported on disease caused by VT (7- and 10-valent) and NVT.

The decline varied, with some studies having a wide confidence interval for the effect estimate (Table 2). Regardless of the vaccine type, serotypes, study design, time period between vaccine roll out to data collection and difference in age groups, the decline ranged from 31.7 to 80.1%. Invasive pneumococcal diseases caused by vaccine serotypes declined significantly, the decline ranged from 35.0 to 92.0%. A much higher decline (55.0–89.0%) was found in children below 24 months of age. One study reported a non-significant percentage increase in relative risk (4.5%, 95%CI = -52.3 to 128.9) [22].

**Table 2:** Effectiveness of PCV in the prevention of IPD among children under five years of age.

	Von Gottberg et al., 2014 [21]		Diawara et al., 2015 [22]	Mackenzie et al., 2016 [24]	Tempia et al., 2015[26]		Nhantumbo et al., 2017 [25]	Von Mollendorf et al., 2016 [27]	Kambire et al., 2018 [23]		Hammitt et al., 2018 [28]
Age & serotypes	Baseline to 2011 Relative difference in rate % (95%CI)	Baseline to 2012 Relative difference in rate % (95%CI)	Relative risk reduction % (95%CI)	Adjusted incidence rate ratio (95%CI)	Relative difference in hospitalization rate 2011	Relative difference in hospitalization rate 2012	Percentage decline of IPD	Odds ratio 2005 vs 2013	Percentage change (95% CI) 2014 vs. 2011–2013	Percentage change (95% CI) 2015 vs. 2011–2013	Adjusted incidence rate ratio (95%CI)
≤ 24 months											
All serotypes	-60(-65 to -56)	-69(-72 to -65)	-60.9(-88.1 to -35.5)	0.45(0.29 to 0.70)	-80.1(-86.2 to -71.8)* -54.8(-72.6 to -27.1)**	-64.0(-72.9 to -52.6)* -66.8(-81.2 to -43.8)**			-49(-60 to -35)	-68(-76 to -57)	
PCV7	-80(-84 to -76)	-89(-92 to -86)	-74.1(-100 to -40.8)	0.17(0.07 to 0.43)	-80.9(-90.9 to -62.9)* -83.2(-94.2 to -59.5)**	-63.8(-79.3 to -39.1)* -91.7(-98.4 to -73.6)**	56.1				
6A	-62(-73 to -47)	-85(-91 to -76)									
1									-60(-81 to -15)	-59(-81 to -14)	
Additional PCV10			-77.7(-93.6 to -22.0)				28.1				
PCV10							84.2				
Additional PCV 13	-22(-39 to -1)	-57(-68 to -42)	-85.2(-100 to 27.9)	0.18(0.06 to 0.56)							
PCV 13				0.18(0.09 to 0.36)	-59.7(-85.9 to +3.4)* -26.3(-74.7 to +107.3)**	+8.8(-94.8 to +57.3)* -63.5(-91.6 to -26.5)**					
NVT	-20(-36 to -0.2)	6(-16 to 23)	28.6 (-61.1 to 100.2)	1.48(0.70 to 3.13)	-82.5(-89.4 to -72.3)* -0.2(-58.0 to +139.2)**	-71.7(-81.1 to -58.5)* +1.2(-96.7 to +58.4)**			-15(-48 to -38)	-69(-84 to -39)	
≥24–59 months											
All serotypes			4.5(-52.3 to 128.9)	0.44(0.25 to 0.75)							
PCV7			-53.7(-81.8 to 128.0)	0.26(0.09 to 0.74)							
Additional PCV10			-3.5(-80.5 to 300.7)								
Additional PCV13			-3.5(-86.4 to 584.7)	0.38(0.17 to 0.85)							
PCV 13				0.32(0.17 to 0.61)							
NVT			285.8(-56.9 to 335.2)	1.27(0.39 to 4.13)							
<60 months											
All types									-41(-55 to -23)	-55(-66 to– 39)	0.32(0.17 to 0.60)
Serotype 1								0.12(0.02 to 0.59)	-30 (-59 to 21)	-25(-56 to 27)	
PCV13									-35(-53 to -10)	-58(-71 to -40)	
PCV10											0.08 (0.03 to 0.22)
NVT									-48(-78 to 21)	-90(-92 to -50)	1.3 (0.65 to 2.64)

*Based on PCR

**Based on culture

CI: confidence interval

For PCV7 serotypes, the overall decline ranged from 56.1 to 91.7% and 53.7 to 74.0% among children less than 24 months and above 24 months, respectively [21,22,24–26]. A stronger decline in PCV7 serotypes was found in studies that used “percentage relative difference in the rate” as the effect estimate. The decline ranged from 63.8 to 91.7% [26].

Two studies estimated the decline in 10-valent vaccine serotypes; in these studies, the percentage relative risk reduction was 84.2 and 92.0% [25,28]. For the three additional 10-valent vaccine specific serotypes (PCV10-nonPCV7 serotypes), significant reductions ranging from 28.1 to 77.7% were recorded among younger children [22,25]. However, among children aged 24–59 months, the decline in IPD was non-significant [22].

Of the included studies, three found a reduction of IPD caused by the 13-valent serotypes, which ranged from 58.0 to 82.0% [24,26]. Furthermore, for children older than two years, the decline was reported to be 68.0% [24]. Two of the included studies estimated a decline for the three additional serotypes present in the 13-valent vaccine and not in the PCV-10 vaccine (3, 6A and 19A). The authors described a significant decline in the rates of IPD for serotypes 3, 6A and 19A. The reported reduction ranged between 22.0 and 82.0% [21,24].

### Relative proportion of vaccine serotypes

**Fig 2:**
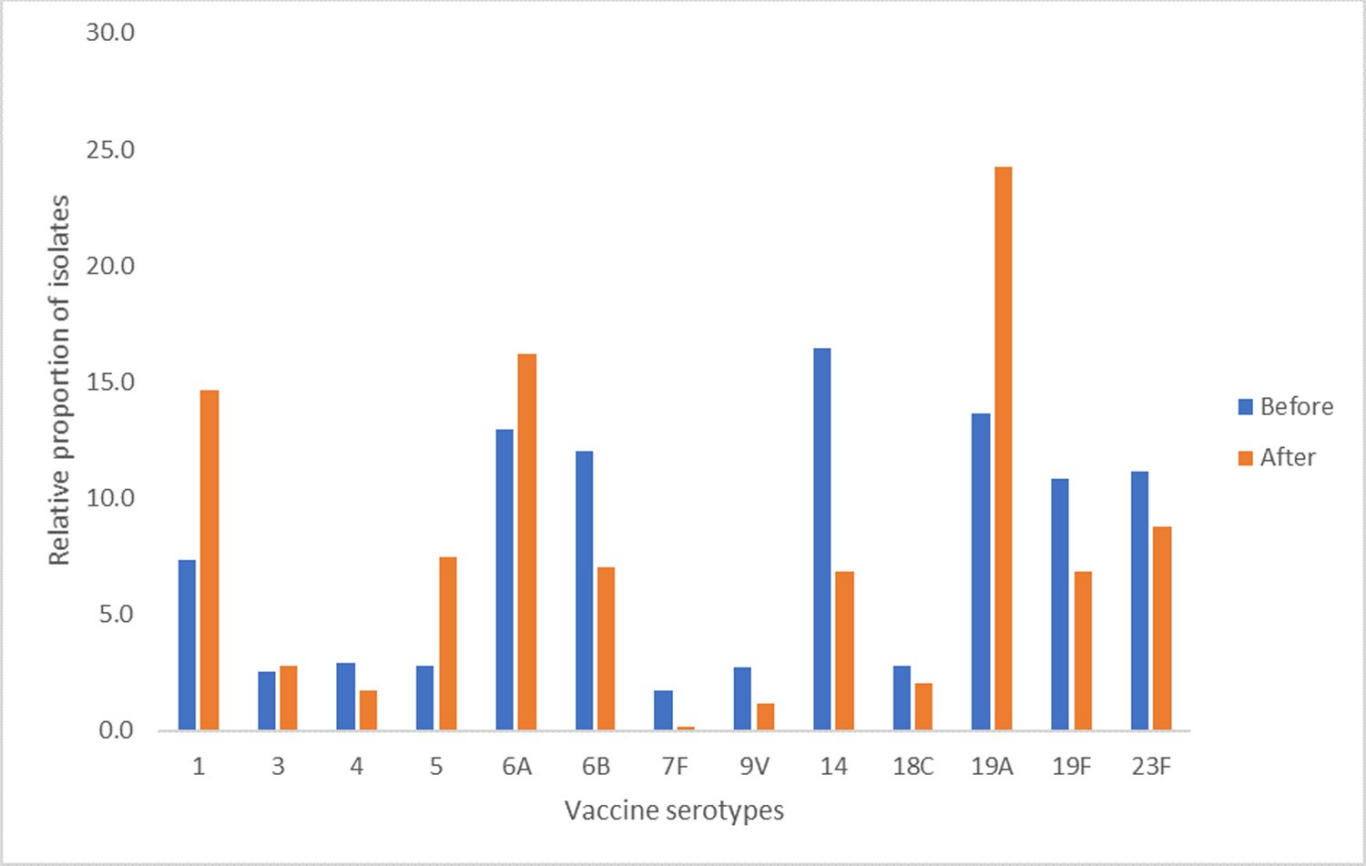
Relative proportion of vaccine serotypes before and after vaccine roll out. Following vaccine roll out, the relative proportions of serotype 1 (7.4%, n = 97 vs 14.6%, n = 100), 5 (2.8%, n = 37 vs 7.5%, n = 51) and 19A (13.7%, n = 180 vs 24.3%, n = 166) doubled compared to the baseline measurement. In contrast, a significant reduction in serotypes 14 (6.9% vs 16.5%), 6B (7.0% vs 12.0%) and 9V (1.2% vs 2.7%) was observed.

Of the total of 2,001 pneumococcal vaccine strains isolated in different countries in different clinical studies conducted in Africa, 1,317 and 684 were isolated before and after the vaccine roll out, respectively. Before the vaccine roll out, the three most common serotypes were 14 (16.5%, n = 271), followed by 19A (13.7%, n = 180) and 6A (13.0%, n = 171) (Fig 2). Following the vaccine roll out, the common vaccine serotypes were 19A (24.3%, n = 166), followed by 6A (16.2%, n = 111) and 1 (14.6%, n = 100).

## Discussion

To the best of our knowledge, this is the first systematic review to describe the post-roll out decline of invasive pneumococcal disease in Africa. Almost ten years post-roll out, data on the effectiveness of the PCV vaccine remain limited in Africa. The inclusion of the few studies from Africa in the global review underestimates the burden of IPD and might not accurately reflect the serotype distribution post-roll out in Africa. Furthermore, some countries lack pre-vaccine data, which makes it difficult to assess vaccine effectiveness.

Although molecular techniques have been used for decades, the common method described in all studies in this review was pneumococcal culture. Across studies, the method used to isolate *S*. *pneumoniae* was not standardized according to the WHO working group recommendation [29]. Although there are no validated methods for direct serotyping of *S*. *pneumoniae* isolates, the culture method poses a risk of underestimating vaccine effectiveness. Previous studies have demonstrated a low sensitivity of culture methods in isolating *S*. *pneumoniae* [30].

The eligibility criteria across the included studies were different, which made it difficult to pool the results to determine vaccine effectiveness. Of note, the design of the included studies was different; whereas three of the included studies were based on national laboratory surveillance, the other five studies were limited to a particular region. Moreover, one study was population-based surveillance with community screening to identify children with IPD, and two were hospital-based prospective IPD case finding. Finally, while six studies analysed the available laboratory samples, one study was a two-time point population-based cross-sectional study (before and after).

Furthermore, different vaccines were used. Studies included in this review were conducted in countries that implemented the 10-valent or 13-valent vaccine. In some countries, such as South Africa, they started with PCV-7, which was later replaced by PCV-13 in 2011. Similarly, Morocco rolled out PCV-13 in 2010, and it was replaced with PCV-10 in 2012.

Most studies identified a decline in the pneumococcal VT post-vaccine roll out in Africa, although with a wider range. The decline shows that the serotypes included in PCV10/13 are the culprit of more than 70% of IPD [31]. Variations in the decline are partly due to the vaccine coverage within countries and among study participants. By the end of 2015, the PCV 10/13 coverage in Africa was estimated at 59%, which was just above the global estimate of 37% [32]. Nevertheless, even in the case of a low vaccination rate among participants, the decline was still observed. A similar finding was reported by Oliveira and colleagues in Latin America [19]. They reported a decline that ranged from 7.4% to 84.6% among hospitalized children with pneumonia, while they found a decline ranging from -14.7% to 66% among the IPD cases [19].

The decline in vaccine serotypes was higher among younger children compared to older children [21,23]. Most of the included studies were conducted immediately or concurrently with vaccine roll out. In some countries, children above the recommended age of vaccination were given a catch-up dose at the time of vaccine roll out [20]. However, in countries such as Gambia, PCV13 was introduced without a catch-up vaccination in children above the recommended age for vaccination [24]. This means that children, especially those above two years of age, were not vaccinated or had not completed the recommended number of doses. Moreover, the decline in younger children is significant because the relative proportion of pneumococcal infection is higher in this age group [1,33].

There have been declines in VT associated with IPD after introduction of the 10-valent and 13-valent PCV vaccines. Routine immunization has also influenced the carriage and association with IPD caused by NVT [34]. We found that, after implementation of PCV10 and 13, the relative proportion of vaccine serotypes was 33.9%. In addition, *S*. *pneumoniae* vaccine-specific serotypes were isolated from 56.8% of children with IPD. These results are consistent (71.2%) with findings from Latin America [35]. The percent of PCV-7 VT (27.4%) in the post-PCV era in Africa is comparable to that reported in Europe [36].

The studies included in this review show a decline in VT causing IPD; nevertheless, approximately half of the IPD cases were caused by NVT, which has been reported previously. For instance, in Denmark, one study reported that NVT caused 91% of IPD in children aged 0–4 years [37]. Moreover, in a study comparing pre- and post-vaccination IPD in Barcelona, Spain, there was an increase from 38 to 72% of NVT in children less than 5 years of age [38]. There is a need for vaccines that are effective against many serotypes. However, data on the serotype distribution should precede the development of new vaccines. For example, efficacy studies with PCV15 have been performed [39,40]; however, the addition of serotype 22F and 33F in PCV15 may not be advantageous for Africa since these two serotypes were only isolated in one study.

Serotype 19A has been shown to be associated with vaccine failure [41,42]. After the introduction of PCV10/13 in Africa, serotype 19A became the predominant vaccine serotype. In the pre-PCV era, serotype 19A was the sixth most prevalent serotype in children with IPD [10]; according to this review, it was the second most common vaccine serotype. The review data show that the relative proportion of the 19A serotype doubled following vaccine roll out. Conversely, our findings are consistent with a recent meta-analysis by Baslells, which also found that serotype 19A (21.6%) was the predominant serotype in children with IPD across regions [35]. These findings are believed to be due to previously reported low opsonophagocytic antibody responses against 19A serotype evoked by PCV-13 [43]. Similar to serotype 19A, the relative proportions of serotype 1 and 5 also doubled following vaccine roll out in African countries. Serotype 1 is currently the third most common vaccine serotype causing IPD among children.

Of interest, serotype 6A is overall the second most common serotype in children with IPD in Africa. Of the three-additional 13-valent serotypes (3, 6A and 19A), serotype 6A and 19A are still the most prevalent serotypes in Africa. However, the relative proportion of serotype 3 was low, which is consistent with a previous review that also reported a low prevalence of serotype 3 compared to 6A and 19A among children with IPD post-PCV era [35].

The results on serotypes distribution should be interpreted with caution. First, approximately half of the included studies were from South Africa, a country that may not reflect other parts of Africa. Secondly, some of the children recruited in these studies had not completed the recommended 3 doses. This demands continuous surveillance and notification of the circulating serotypes in different African countries. Furthermore, to draw valid conclusions on effectiveness of PCV vaccination in Africa, more and larger studies are required.

### Limitations

Generally, there is a lack of uniformity in the published vaccination studies that have been conducted in Africa. The study designs were different, some being population-based surveillance while others were laboratory surveillance studies, and the methods used to identify *S*. *pneumoniae* were also different. This heterogeneity of the different studies and the mediocre quality of some studies included in this review made it difficult to conduct a pooled analysis. One of these studies had a small sample size, which might have contributed to the observed wide confidence intervals in the relative differences. Additionally, no statistical tests were performed to provide p-values for the pre-to-post changes. Furthermore, a proportion of the children included in the studies had not received the vaccine or had not received all of the recommended doses. As a result, we might have underestimated the potential decline of IPD that can be achieved by PCV vaccination. This review included studies with different designs and different methods of pneumococcal detection (culture, latex and PCR), which has influenced the reported decline in VT post-PCV roll out. In some of the included studies, no information was available on the exact number of serotypes included in the analyses. In such cases, the number of serotypes was calculated from proportions and sometimes extracted from figures. In addition, information on the total number of children under the age of 5 at risk was not available in some studies and was estimated from the incidence or rates. While NVT contributed significantly to IPD morbidity and mortality, it was not possible to conclude which NVT was most common as there was no uniformity in the reporting of these serotypes. We suggest performing further studies in other (currently understudied) parts of Africa to better understand the effectiveness of PCV vaccination. More importantly, there is a strong demand for more uniformly conducted vaccine effectiveness studies measuring pneumococcal VT and NVT in IPD and colonization to strengthen the conclusions on the effect of PCV implementation in Africa. Lastly, it is unlikely that publications about relevant vaccine studies were missed in our search as we selected articles in English. However, this limitation cannot be fully excluded considering the high number of francophone countries, particularly in west Africa.

## Conclusion

After the introduction of PCV in Africa, a decline was observed in pneumococcal vaccine serotypes among children below the age of 5 years. The strongest effects were measured in children less than two years of age. Remarkably, the relative proportion of the three serotypes (1, 5 and 19A) of the 13-valent vaccine doubled following vaccine roll out. Serotypes 6A and 19A were most common among children with IPD. More and larger studies in different parts of Africa are needed to thoroughly assess the effectiveness of PCV vaccination.

### Funding source

This research was supported by the Fogarty International Center of the National Institutes of Health under Award Number D43TW010138. Additionally, this work was supported partly by the German Academic Exchange Service (Deutscher Akademischer Austauschdienst-DAAD). James Samwel Ngocho is a medical education partnership junior faculty fellow and DAAD fellow. The funders had no role in study design, data collection and analysis, decision to publish, or preparation of the manuscript.

## Supporting information

S1 PRISMA checklist (DOC)

S1 Search strategy (PubMed & Scopus) (DOCX)

S1 Risk of bias assessment for effectiveness (DOCX)

S1 Effectiveness data (XLSX)

S1 Proportion of vaccine serotypes data (XLSX)
