# Supplementary material for: Effectiveness of pneumococcal conjugate vaccines against invasive pneumococcal disease among children under five years of age in Africa: A systematic review
Source: PLoS One. 2019 Feb 19;14(2):e0212295. doi: 10.1371/journal.pone.0212295 (PMC6380553; doi:10.1371/journal.pone.0212295)
Supplement: S1 Search strategy (PubMed & Scopus) — (DOCX) [file pone.0212295.s002.docx]

**Appendix 1**

Keywords; Disease burden, *S. pneumoniae*, Serotypes and 10/13-valent vaccine

Synonyms

Disease burden

- Prevalence
- Prevalences
- Frequency
- Proportion
- Incidence
- epidemiology

*S. pneumoniae*

- Pneumococcus
- Streptococcus pneumoniae
- Pneumococcal
- Pneumococci

Serotype

- Serogroups
- Serotypes
- Serovar
- Serovars

10/13-valent vaccine

- pneumococcal vaccine
- 10/13 valent vaccine
- PCV10/13
- Pneumococcal conjugate vaccine
- Prevenar10/13
- PCV10/13 vaccine
- PCV-13/10 vaccine
- prevenar 13
- prevnar 10/13
- 10/13 valent pneumococcal vaccine

**PubMed search strategy**

Search

1. ("epidemiology"[Subheading] OR "epidemiology"[All Fields] OR "prevalence"[All Fields] OR "prevalence"[MeSH Terms]) OR ("prevalence"[MeSH Terms] OR "prevalence"[All Fields] OR "prevalences"[All Fields]) OR burden[All Fields] OR ("epidemiology"[Subheading] OR "epidemiology"[All Fields] OR "frequency"[All Fields] OR "epidemiology"[MeSH Terms] OR "frequency"[All Fields]) OR proportion[All Fields] OR ("epidemiology"[Subheading] OR "epidemiology"[All Fields] OR "incidence"[All Fields] OR "incidence"[MeSH Terms])
2. ("streptococcus pneumoniae"[MeSH Terms] OR ("streptococcus"[All Fields] AND "pneumoniae"[All Fields]) OR "streptococcus pneumoniae"[All Fields] OR "s pneumoniae"[All Fields]) OR (("streptococcus"[MeSH Terms] OR "streptococcus"[All Fields] OR "streptococcal"[All Fields]) AND ("pneumonia"[MeSH Terms] OR "pneumonia"[All Fields] OR "pneumoniae"[All Fields])) OR ("streptococcus pneumoniae"[MeSH Terms] OR ("streptococcus"[All Fields] AND "pneumoniae"[All Fields]) OR "streptococcus pneumoniae"[All Fields] OR "pneumococcus"[All Fields]) OR ("streptococcus pneumoniae"[MeSH Terms] OR ("streptococcus"[All Fields] AND "pneumoniae"[All Fields]) OR "streptococcus pneumoniae"[All Fields])
3. ("serogroup"[MeSH Terms] OR "serogroup"[All Fields] OR "serogroups"[All Fields]) OR ("serogroup"[MeSH Terms] OR "serogroup"[All Fields] OR "serotype"[All Fields]) OR ("serogroup"[MeSH Terms] OR "serogroup"[All Fields] OR "serotypes"[All Fields]) OR ("serogroup"[MeSH Terms] OR "serogroup"[All Fields] OR "serovar"[All Fields]) OR ("serogroup"[MeSH Terms] OR "serogroup"[All Fields] OR "serovars"[All Fields])
4. ("pneumococcal vaccines"[MeSH Terms] OR ("pneumococcal"[All Fields] AND "vaccines"[All Fields]) OR "pneumococcal vaccines"[All Fields] OR ("pneumococcal"[All Fields] AND "vaccine"[All Fields]) OR "pneumococcal vaccine"[All Fields]) OR (13-valent[All Fields] AND ("vaccines"[MeSH Terms] OR "vaccines"[All Fields] OR "vaccine"[All Fields])) OR (13[All Fields] AND valent[All Fields] AND ("vaccines"[MeSH Terms] OR "vaccines"[All Fields] OR "vaccine"[All Fields])) OR PCV13[All Fields] OR PCV10[All Fields] OR (("pneumococcal vaccines"[MeSH Terms] OR ("pneumococcal"[All Fields] AND "vaccines"[All Fields]) OR "pneumococcal vaccines"[All Fields] OR "pneumococcal"[All Fields]) AND ("vaccines, conjugate"[MeSH Terms] OR ("vaccines"[All Fields] AND "conjugate"[All Fields]) OR "conjugate vaccines"[All Fields] OR ("conjugate"[All Fields] AND "vaccine"[All Fields]) OR "conjugate vaccine"[All Fields])) OR "prevenar13"[All Fields] OR ("prevenar13"[All Fields] OR "pcv10 vaccine"[All Fields]) OR ("prevenar13"[All Fields] OR "pcv13 vaccine"[All Fields]) OR ("prevenar10"[All Fields] OR "pcv 13 vaccine"[All Fields]) OR "pcv 10 vaccine"[All Fields]) OR ("prevenar13"[All Fields] OR "prevenar 13"[All Fields]) OR ("prevenar13"[All Fields] OR "prevnar 13"[All Fields]) OR ("13-valent pneumococcal vaccine"[Supplementary Concept] OR "13-valent pneumococcal vaccine"[All Fields] OR "13 valent pneumococcal vaccine"[All Fields]) OR "10 valent pneumococcal vaccine"[All Fields])
5. (#1 AND #2 AND #3 AND #4)

**Scopus search strategy**

Search

1. **TITLE-ABS-KEY (prevalence OR prevalences OR epidemiology OR frequency OR incidence OR proportion OR budren**)
2. TITLE-ABS-KEY (**"S. pneumoniae" OR Pneumococcus OR "Streptococcus pneumoniae" OR Pneumococcal OR Pneumococci**)
3. TITLE-ABS-KEY (**"pneumococcal conjugate vaccine" or "13-valent vaccine" or Pcv13 or "13 valent pneumococcal vaccine"**) or **"10-valent vaccine or Pcv13 or 10 valent pneumococcal vaccine**
4. TITLE-ABS-KEY (**serotype* OR serogroup* OR serovar***)
5. #1 AND #2
6. #5 AND #3
7. #6 AND #4
