# Supplementary material for: Effectiveness of pneumococcal conjugate vaccines against invasive pneumococcal disease among children under five years of age in Africa: A systematic review
Source: PLoS One. 2019 Feb 19;14(2):e0212295. doi: 10.1371/journal.pone.0212295 (PMC6380553; doi:10.1371/journal.pone.0212295)
Supplement: S1 Risk of bias assessment for effectiveness — (DOCX) [file pone.0212295.s003.docx]

**Table**: Quality assessment for before-after (pre-post) studies with No control group

| Criteria | Studies | | | | | | | |
| --- | --- | --- | --- | --- | --- | --- | --- | --- |
|  | Von Gottberg et al., 2014 | Diawara et al., 2015 | Mackenzie et al., 2016 | Von Mollendorf et al., 2016 | Tempai et al., 2015 | Nhantumbo et al., 2017 | Hammitt et al., 2018 | Kambire et al., 2018 |
| Was the study question or objective clearly stated? | Yes | Yes | Yes | Yes | Yes | Yes | Yes | Yes |
| Were eligibility/selection criteria for the study population prespecified and clearly described? | Yes | Yes | Yes | Yes | Yes | Yes | Yes | Yes |
| Were the participants in the study representative of those who would be eligible for the test/service/intervention in the general or clinical population of interest? | Yes | Yes | Yes | Yes | Yes | Yes | Yes | Yes |
| Was the sample size sufficiently large to provide confidence in the findings? | Yes | No | Yes | Yes | Yes | Yes | Yes | Yes |
| Was the test/service/intervention clearly described and delivered consistently across the study population? | CD | CD | CD | CD | CD | CD | CD | CD |
| Were the outcome measures prespecified, clearly defined, valid, reliable, and assessed consistently across all study participants? | Yes | Yes | Yes | Yes | Yes | Yes | Yes | Yes |
| Were the people assessing the outcomes blinded to the participants' exposures/interventions? | No | No | No | No | No | No | No | No |
| Was the loss to follow-up after baseline 20% or less? Were those lost to follow-up accounted for in the analysis? | NA | NA | NA | NA | NA | NA | NA | NA |
| Did the statistical methods examine changes in outcome measures from before to after the intervention? Were statistical tests done that provided p values for the pre-to-post changes? | Yes | Yes | Yes | Yes | Yes | No | Yes | Yes |
| Were outcome measures of interest taken multiple times before the intervention and multiple times after the intervention (i.e., did they use an interrupted time-series design)? | No | Yes | Yes | No | No | No | Yes | Yes |
| If the intervention was conducted at a group level (e.g., a whole hospital, a community, etc.) did the statistical analysis take into account the use of individual-level data to determine effects at the group level? | NA | NA | NA | NA | NA | NA | Na | NA |
| **Quality Rating (Good, Fair, or Poor)** | Good | Fair | Good | Good | Good | Fair | Good | Good |
| CD, cannot determine; NA, not applicable; NR, not reported | | | | | | |  |  |
